# miR-20a regulates sensitivity of colorectal cancer cells to NK cells by targeting MICA

**DOI:** 10.1042/BSR20180695

**Published:** 2019-07-02

**Authors:** Siwen Tang, Hongyu Fu, Qihua Xu, Ying Zhou

**Affiliations:** 1 Department of General Surgery, Second Hospital of Heilongjiang Province, Harbin 150000, Heilongjiang, China; 2 Department of Gastroenterology, Changhai Hospital, Second Military Medical University, Shanghai 200082, China; 3 Gastroenterology, Seventh People’s Hospital of Shanghai University of Traditional Chinese Medicine, Shanghai 200137, China

**Keywords:** CRC, MICA, microRNA, miR-20a, Natural Killer cells, NKG2D

## Abstract

Colorectal cancer (CRC) is one of the leading cancer-related causes of deaths in the world. Recently, microRNAs have been reported to regulate the tumor growth, invasion and the immunosuppression. In the present study, we found that miR-20a was increased in human CRC specimens compared with the healthy normal tissues. However, miR-20a overexpression and knockdown did not impair the CRC cell growth *in vitro*. Our results indicated that CD107a^+^ NK cells are increased in CRC group. Furthermore, cytotoxicity assays demonstrated that miR-20a knockdown promoted the CRC cells sensitive to NK cells, whereas miR-20a overexpression showed the opposite results. Our results suggest that the regulation of NK cells by miR-20a depends on NKG2D. Luciferase reporter assays revealed that the NKG2D ligand Major Histocompatibility Complex (MHC) class I-related chain genes A (MICA) is the direct target of miR-20a. Flow cytometry showed the MICA protein level is significantly reduced in miR-20a-overexpressing CRC cells and increased in miR-20a knockdown CRC cells. Taken together, our results suggest that miR-20a regulates sensitivity of CRC cells to NK cells by targeting MICA.

## Introduction

Colorectal cancer (CRC) is one of the most common tumors worldwide and has poor prognosis despite advanced methods in diagnosis and treatment [1,2]. The distant metastasis is caused of high mortality of CRC. From the diagnosis of stage I and II cases, surgical resection is considered the mainstay treatment, with a 5-year survival rate of 80% [3]. The standard management of advanced CRC has historically focused on multi-agent chemotherapy. More recently, development of immunotherapy, including monoclonal antibody treatment and immune cell immunotherapy, led to their application in metastatic patients [4,5]. It has been reported that the immune response in CRC was associated with clinical outcomes [6]. It is all known that CRC cells remain susceptible to T cell-mediated killing despite prior exposure to chemotherapy [7]. Innate immunity is involved in tumor immunosurveillance and NK cells play very important roles in the clearance of tumor cells, which is displayed with reduced Major Histocompatibility Complex (MHC) I expression [8].

MicroRNAs are short non-coding RNAs that negatively regulate gene expression by inhibiting their target mRNA translation or accelerating mRNA degradation. It is reported that microRNAs have important roles in many biological processes, including cell growth, cell apoptosis, cell differentiation, immune responses and also tumor progression [9–11]. microRNA-20a has been shown to play functional roles in many types of cancers, including nasopharyngeal cancer cells [12], breast carcinoma [13,14] and gastric cancer [15,16]. However, it has not been reported whether miR-20a also worked as a functional factor in CRC.

In the present study, we first notified that miR-20a level was significantly increased in CRC tissues. However, miR-20a overexpression or knockdown did not affect the CRC cell growth. It is very interesting that miR-20a overexpression reduced the sensitivity of CRC cells to cytotoxic NK cells, whereas miR-20a knockdown CRC cells showed the opposite phenotype. Additionally, luciferase reporter assays revealed that NKG2D ligand MHC class I-related chain genes A (MICA) was the direct target of miR-20a in CRC cells. Taken together, we propose that miR-20a may have an oncogenic role in CRC progression by targeting MICA to regulate NK cell activity.

## Materials and methods

### Clinical samples

Patients who were diagnosed with primary CRC and confirmed by pathologic examinations, were recruited with written informed consent to the present study. The present study was approved by the Medical Ethics Committee of Seventh People’s Hospital of Shanghai University of Traditional Chinese Medicine. For human samples, the research has been carried out in accordance with the World Medical Association Declaration of Helsinki. Tumor tissues were collected immediately after resection and were stored in liquid nitrogen before further use.

### Cell culture

HEK293T, SW480 and HCT116 was obtained from the American Type Culture Collection (ATCC). The cells were cultured in DMEM (HEK293T) or RPMI 1640 (other cell lines) culture medium supplemented with 10% fetal bovine serum, 2 mM l-glutamine, 100 mg/ml streptomycin and 100 U/ml penicillin in an incubator with a humidified atmosphere and 5% CO_2_ at 37°C.

### Quantitative PCR

Total RNAs were extracted from cancer cells or CRC tumor tissues by using RNAiso Plus reagent (Takara Biotechnology Co., Ltd, Dalian). qRT-PCR of miR-20a was carried out using the TaqMan has-miR-20a Assay (Assay ID: 000580; Mature sequence: UAAAGUGCUUAUAGUGCAGGUAG) (Thermo Fisher Scientific, Waltham, MA, U.S.A.). Transcripts were quantitated by real-time PCR and normalized to the amount of U6 expression. For MICA mRNA expression analysis, first strand cDNA was synthesized by using cDNA synthesis kit (Takara) according to the manufacturer’s instructions. Their expression at mRNA level were detected by using Syber Green PCR mastermix (Applied Biosystems). The primers of MICA and Actin mRNA were listed as follows: Actin Forward: 5′-AGCCTCAAGATCATCAGCAATGCC-3′, Actin Reverse: 5′-TGTGGTCATGAGTCCTTCCACGAT-3′; MICA Forward: 5′-ACACCCAGCAGTGGGGGGAT-3′, MICA Reverse: 5′-GCAGGGAATTGAATCCCAGCT-3′.

### MTT assay

CRC cells were seeded in 96-well plates (4 × 10^3^ cells per well) at 37°C in an incubator containing 5% CO_2_. Cells were transfected with miR-20a mimics/antagomir-20a. Cell viability was tested by using 3-(4,5-Dimethyl-2-thiazolyl)-2,5-diphenyl-2H-tetrazolium bromide (MTT, Sigma) assay at 0, 12, 24, 36, 48 h after treatment. Briefly, cells were incubated with MTT at a final concentration of 0.5 mg/ml for 4 h. The supernatant was discarded, and the precipitated formazan was dissolved in dimethyl sulfoxide. Absorbance was measured at 490 nm with microplate reader (Molecular Devices, i3).

### Luciferase reporter assay

The DNA oligonucleotide and the pMiR-Reporter Vector were used to build the luciferase report vectors (pMiR-MCIA-WT and pMiR-MCIA-Mutant, Figure 2A). HEK293 cells were co-transfected with pMiR-MCIA-WT or pMiR-MICA-Mutant and miR-20a mimics or negative control (NC). A *Renilla* luciferase-expressing plasmid pRL-TK (Promega) used as control was also co-transfected. Cells were harvested and luciferase activity was determined using the Dual Luciferase Reporter Assay Kit (Promega) at 24 h after transfection. The results are expressed as relative luciferase activity (firefly luciferase/*Renilla* luciferase).

### Flow cytometry

Direct immunofluorescence was performed on PBMC or NK cells using specific mAbs and isotype-matched mAbs. Cells were washed with PBS and stained for 20 min at 4°C. NK cells, gated on CD3^−^CD56^+^ cells in lymphocyte subset were analyzed for the expression of CD107a (BD Biosciences, CD3: 555335, CD56: 561903, CD107a: 553793). The expression of MICA on CRC cells were also examined by FACS. CRC cells were transfected with miR-20a, miR-20a antagonist or miR-NC. These cells were collected for staining with MICA (Biolegend, clone:6D4, 320906). Cells were collected on an FACSCalibur flow cytometry. These data were analyzed using FlowJo software.

### Cell cytotoxicity assay

Cell cytotoxicity was determined by flow cytometry using the CRC cell lines SW480 and HCT116 cells as target cells, whereas NK cells and NK-depleted PBMCs as the effect cells. NK cells were isolated from healthy PBMCs by magnetic sorting and then incubated with 500 U/ml hrIL-2 for 16 h. Human NK cells are purified by human NK cell isolation kit (Miltenyi Biotec). The target cells were labeled with CFSE and then co-cultured with effect cells for 16 h. These cells were subjected to 7-AAD staining and acquired by flow cytometry for testing the killing frequency of target cells. In addition, we also blocked NKG2D by antibody (αNKG2D, Thermo Fisher Scientific, 14-5878-82) and then determined the NK cell cytotoxicity. For CRC cell surface MICA expression, cells were stained with APC–conjugated mouse anti-human MICA or amount of corresponding isotype control (BD Biosciences).

### Statistical analysis

All analyses were performed using the statistical software GraphPad Prism. Data were presented as mean ± S.E.M. Group comparison was performed by Student’s *t* test. *P*-value <0.05 was considered as significant difference. *, **, and *** denote significance at 0.05, 0.01 and 0.001 levels, respectively.

## Results

### CRC patients showed altered expression of miR-20a and proportions of NK cells

**Figure 1:**
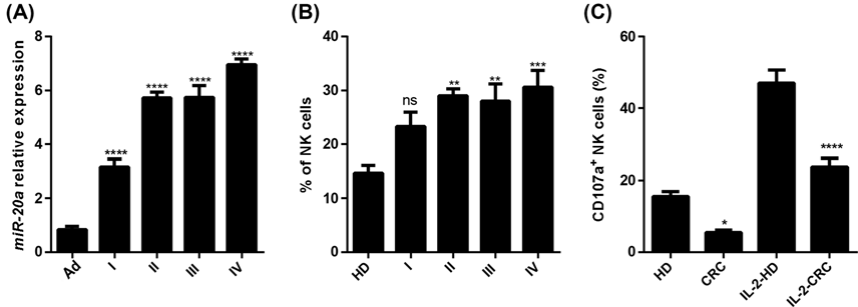
miR-20a expression was increased in CRC (**A**) The expression of miR-20a at different stages of CRC tumor tissues and CRC tumor adjacent tissues was examined by qRT-PCR. (**B**) NK proportions in lymphocytes population of different stages of CRC patients and healthy donors. (**C**) CD107a^+^ NK cells from HD and CRC patients pre-stimulated were examined by FACS with or without IL-2. qRT-PCR results were normalized by β-actin. All data were shown as mean ± S.E.M. from three independent experiments. * represents *P*<0.05, ** represents *P*<0.01, *** represents *P*<0.001, **** represents *P*<0.0001, ns represents no significance.

In an observational and exploratory study, 35 CRC patients were enrolled, 14 from I–II stages without lymph node invasion and 21 patients with lymph node or metastatic dissemination from III–IV stages. Twelve health samples were analyzed as the control. microRNAs have been reported to act as functional roles in various biological processes such as tumor cell proliferation, apoptosis, invasion and lymphocytes infiltration. To investigate whether there are some microRNAs that could regulate the tumor progression by affecting immune cell function, the expression of microRNA-20a was determined by quantitative PCR analysis. The results showed that the expression of miR-20a was significantly increased in CRC patients, even in I stage CRC patients (Figure 1A). Phenotypic analysis from CRC patients displayed a significant increase in NK cells proportions among the lymphocyte subset as compared with adjacent tissues (Figure 1B). Nevertheless, the frequency of CD107a NK cells, a NK cell degranulation marker, from CRC patients was lower than those achieved from healthy control patients following IL-2 activation (Figure 1C). In summary, our results revealed that miR-20a expression are increased in CRC and the function of NK cells from CRC is altered.

### MICA was the direct target of miR-20a in CRC cells

**Figure 2:**
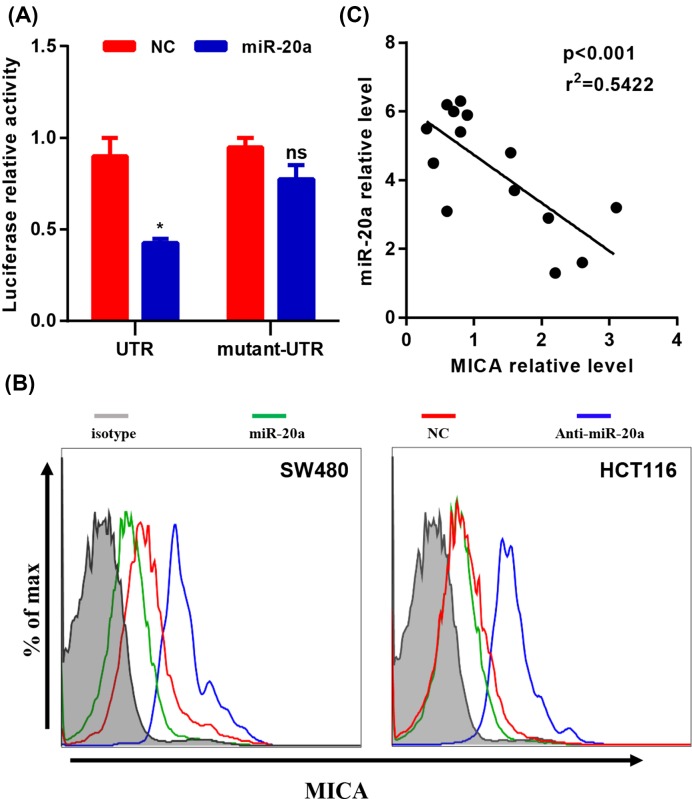
MICA is the target of miR-20a in CRC cells (**A**) HEK-293 cells were co-transfected with either 100 nM miR-20a mimics or control oligos (NC) and 200 ng plasmid carrying either wild-type or mutant 3′-UTR of MICA. The relative firefly luciferase activity normalized with *Renilla* luciferase was measured 48 h later. (**B**) The MICA protein level in miR-20a overexpressing or knockdown CRC cells were determined by flow cytometry. (**C**) The correlation between miR-20a mRNA level and MICA mRNA level were examined by RT-PCR. All data were shown as mean ± S.E.M. from three independent experiments. * represents *P*<0.05 and ns represents no significance. Abbreviation: 3′-UTR, 3′-untranslated region.

To investigate the target of miR-20a in CRCs, we explored if there were potential binding sites of miR-20a. By using microRNA target prediction software (www.microrna.org) [31], we notified that NKG2D ligand MICA was a potent candidate. To verify whether MICA is the target of miR-20a, luciferase reporter assays were performed. Relative luciferase activity showed that MICA was the direct target of miR-20a in CRC cells (Figure 2A). In addition, flow cytometry also showed that miR-20a overexpression suppressed the cell surface MICA protein level, whereas miR-20a knockdown increased surface MICA protein level (Figure 2B). To further confirm whether MICA level was regulated by miR-20a, clinical samples were subjected to quantitative PCR analysis. The results suggested that the MICA mRNA level showed the negative correlation with miR-20a level in these samples (Figure 2C). Taken together, our results suggested that NKG2D ligand MICA was the direct target of miR-20a, and these results also suggest that miR-20a might regulate the NK cell function to promote cancer cell escape from NK cell killing.

### miR-20a level regulated CRC cell sensitivity to NK cells

According to the results we have talked above, it is worth investigating the correlations between increasingly expressed miR-20a and NK cell dysfunction. First, we altered miR-20a level in two CRC cells, SW480 and HCT116 cells. These cells are transfected with miR-20a and miR-20a antagonist (anti-miR-20a) (Figure 4A). First, we examined the cell growth after miR-20a and its antagonist transfection. MTT assays revealed that miR-20a expression did not alter these two CRC cell growth (Figure 4B). We next evaluated whether miR-20a was responsible for the sensitivity of CRC cells to NK cells. We isolated NK cells from healthy PBMCs by magnetic sorting and then expanded these NK cells by IL-2 for 24 h. To further explore the role of miR-20a in CRC cells, NK cells were co-cultured with miR-20a overexpressing CRC cells or miR-20a knockdown cells at different effect:target cells (E:T) ratios. NK cytotoxicity against miR-20a overexpressing cells were significantly lower than that against control cells, whereas increased NK cytotoxicity was demonstrated in miR-20a knockdown cells when compared with control cells (Figure 4C). These results indicated that the expression of miR-20a may indirectly regulate NK cell cytotoxicity.

**Figure 3:**
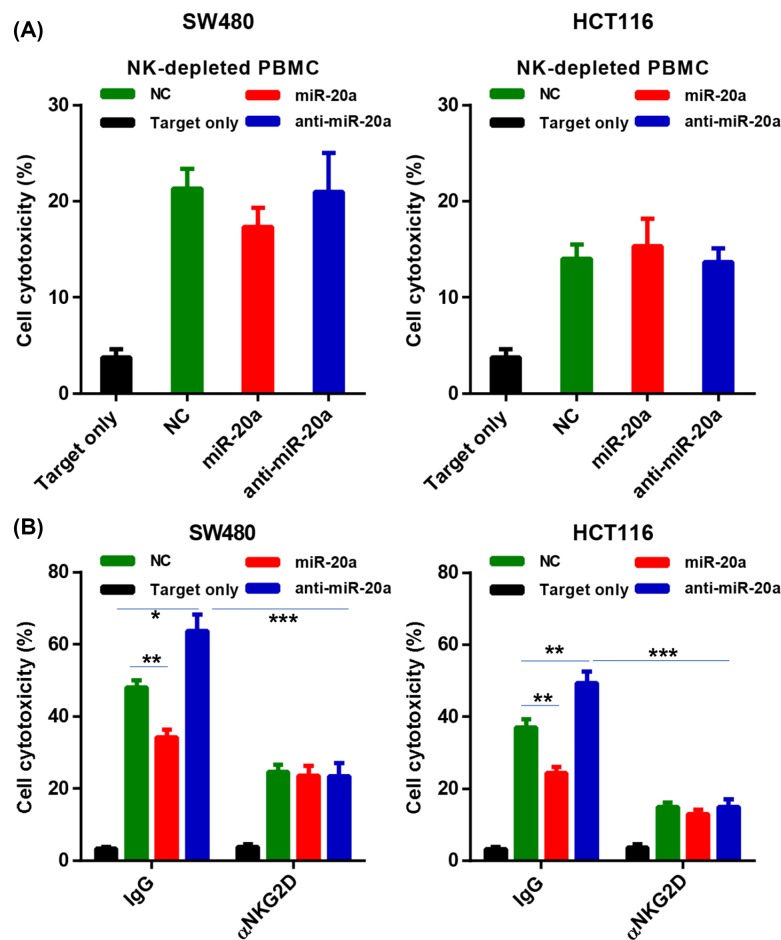
miR-20a regulates CRC cells sensitivity to NK cells depending on NKG2D signaling (**A**) Depletion of NK cells markedly abolished the cell cytotoxicity. CD56^+^ NK cells were depleted from PBMCs by magnetic sorting. NK cell-depleted PBMCs were then co-cultured with miR-20a overexpressing and knockdown of CRC cells at an E:T ratio of 50:1 for 16 h. (**B**) NK cells were co-cultured with miR-20a overexpressing and knockdown CRC cells at an E:T ratio of 50:1 in the presence or absence of NKG2D blocking antibody for 16 h. The specific kill of target cells were examined by FACS. All data were shown as mean ± S.E.M. from three independent experiments. * represents *P*<0.05, ** represents *P*<0.01, *** represents *P*<0.001 and ns represents no significance.

To extensively examine how miR-20a affected NK cell cytotoxicity, we next co-culture these CRC cells with NK-depleted PBMC cells. CD56 NK cells were depleted from healthy PBMCs by magnetic sorting. Upon NK cell depletion, the decreased cytotoxicity against miR-20a overexpressed CRC cells was abolished, whereas the increased cytotoxicity against miR-20a knockdown CRC cells was restored (Figure 3A). Furthermore, we also blocked NK activation receptor NKG2D by antibody in the NK:CRC cell co-culture system. Flow cytometry analysis showed that NKG2D blocking significantly restored the NK cytotoxicity against both miR-20a overexpression CRC cell and miR-20a knockdown CRC cells (Figure 3B). This result indicated that miR-20a regulated NK cell dysfunction was NKG2D dependent.

**Figure 4:**
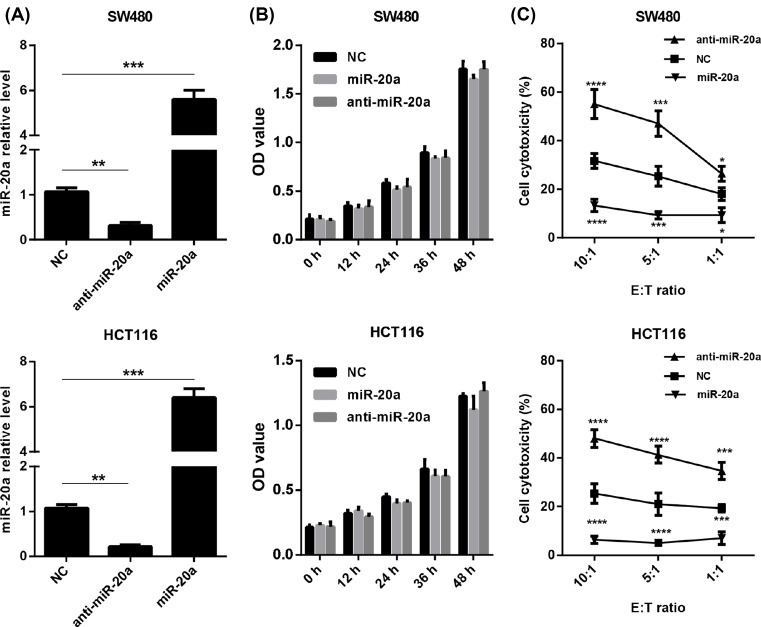
miR-20a overexpressing CRC cells were resistant to NK cells. (**A**) Relative miR-20a expression in CRC cells, which were transfected with miR-20a mimics and anti-miR-20a mimics, was examined by qRT-PCR. (**B**) Cell viability of CRC cells, transfected with miR-20a mimics and miR-20a antagomir was analyzed at indicated time points. (**C**) Lysis of the CRC cells by NK cells from heathy donors. The percentage of specific lysis of CRC cells by NK cells was determined at different E:T ratios. All data were shown as mean ± S.E.M. from three independent experiments. * represents *P*<0.05, ** represents *P*<0.01, *** represents *P*<0.001 and ns represents no significance.

## Discussion

MicroRNAs (miRNAs) are small molecular non-coding RNAs that are 18–25 nucleotides in length [17]. Mature miRNA mainly degrades or inhibits the transcription of mRNA or translation of proteins by binding to the 3′-untranslated region (3′-UTR) of target genes to regulate many aspects of tumor cell function including regulation of the cell cycle, proliferation and apoptosis [18,19]. Previous studies have reported that miR-20a is not only related to tumor development but is also involved in tumorigenesis [20,21]. However, the role of miR-20a in CRC has never been reported. In the present study, we demonstrated that miR-20a regulates NKG2D ligand MICA expression in CRC cells to promote tumor growth.

Although previous studies have revealed that miR-20a directly regulates many types of tumor cells growth and metastasis [13,22–24], these studies mainly focused on the impact of miR-20a in tumor cell themselves. These results promote us to investigate the role of miR-20a in CRC growth. In the present study, our results revealed there is no impact of miR-20a on the growth of CRC cells. However, quantitative PCR analysis showed the miR-20a level are increased in CRC tumor tissues. In the present study, we also found that the activation of NK cells was reduced in CRC patients. This result suggests that miR-20a may indirectly regulate the activation of NK cells. To evaluate the impact of miR-20a on NK cell function, we overexpressed or knocked down miR-20a in CRC cancer cells and then mixed these cells with NK cells. Flow cytometry analysis indicated that miR-20a overexpression significantly reduced the CRC cell sensitivity to NK cells, whereas miR-20a knockdown showed the opposite results.

The activating receptor NKG2D and its ligands play important roles in NK cell-mediated immune responses to tumors [25,26]. In humans, the NKG2D ligands are MICA, MICB, and six members of the ULBP family. In mice, ligands can be divided into three subgroups: five different isoforms of the Rae1-family (α–ε), MULT1, and three different isoforms of H60 family [27]. The MICAs are a new family of proteins encoded within the human HLA class I genes [28,29]. It has been reported to be the activating ligand of NK cells. In our study, miR-20a does not affect the growth of CRC tumor cells, but it can regulate the CRC cell sensitivity to NK cells. This promotes us to ask if miR-20a can regulate the NKG2D ligand expression on CRC cells. Bioinformatics analysis and dual luciferase reporter assays showed miR-20a can directly bind to MICA 3′-UTR. FACS analysis also showed the MICA expression reduced in miR-20a overexpressing CRC cells and its expression increased in miR-20a knockdown CRC cells. And also, MICA levels showed the negative correlation to miR-20a in CRC tumor tissues. These results indicated miR-20a suppressed NKG2D ligand MICA expression on CRC cells to promote CRC growth. There are also some papers reporting that drugs can regulate tumor cell microRNA processing [30] and these tumor cells may display increased resistance to NK cell killing.

In summary, the present study revealed miR-20a regulates CRC growth by suppressing MICA expression on CRC cells and therefore escape NK cell killing. Taken together, these data indicate a new role for miR-20a in CRC growth and provide novel insights into MICA modulation during cancer immune surveillance.

## Informed Consent

Informed consent was obtained from all individual participants included in the study.

## Abbreviations

APCs: antigen presenting cells
CRC: colorectal cancer
MHC: major histocompatibility complex
MICA: MHC class I-related chain genes A
MICB: MHC class I-related chain genes B
MTT: 3-(4,5-Dimethyl-2-thiazolyl)-2,5-diphenyl-2H-tetrazolium bromide
NC: negative control
NK: natural killer cells
NKG2D: natural killer group member 2D
3′-UTR: 3′-untranslated region
